# Experimental realization of a Rydberg optical Feshbach resonance in a quantum many-body system

**DOI:** 10.1038/s41467-018-04684-w

**Published:** 2018-06-08

**Authors:** O. Thomas, C. Lippe, T. Eichert, H. Ott

**Affiliations:** 1 Department of Physics and Research Center OPTIMAS, Technische Universität Kaiserslautern, Erwin-Schrödinger-Straße 46, 67663 Kaiserslautern, Germany; 2 Graduate School Materials Science in Mainz, Staudinger Weg 9, 55128 Mainz, Germany

## Abstract

Feshbach resonances are a powerful tool to tune the interaction in an ultracold atomic gas. The commonly used magnetic Feshbach resonances are specific for each species and are restricted with respect to their temporal and spatial modulation. Optical Feshbach resonances are an alternative which can overcome this limitation. Here, we show that ultra-long-range Rydberg molecules can be used to implement an optical Feshbach resonance. Tuning the on-site interaction of a degenerate Bose gas in a 3D optical lattice, we demonstrate a similar performance compared to recent realizations of optical Feshbach resonances using intercombination transitions. Our results open up a class of optical Feshbach resonances with a plenitude of available lines for many atomic species and the possibility to further increase the performance by carefully selecting the underlying Rydberg state.

## Abstract (web summary)

Ultracold Rydberg molecules have characteristic properties that can be exploited for quantum control and applications. Here the authors demonstrate a Rydberg optical Feshbach resonance, which is based on ultra-long-range Rydberg molecules.

## Introduction

Feshbach resonances in ultracold atomic gases have led to important advances in many-body physics. They did not only enable ground breaking work in the crossover regime from a Bose–Einstein condensate of molecules to a Bardeen–Cooper–Schrieffer type superfluid of fermionic pairs^1^, but are also widely used for the association of ultracold molecules^2^, leading to the formation of molecular Bose–Einstein condensates^3,4^ and ultracold dipolar molecular systems^5^. Most experiments so far used magnetic Feshbach resonances, which are a powerful and well-established tool to change the interaction of an atomic species^2^. However, they often require ultrastable and strong magnetic fields and are only available for certain atomic species and magnetic sublevels. In addition, magnetic Feshbach resonances are limited with respect to their spatial and temporal modulation. The latter can be overcome by using an optically switched magnetic Feshbach resonance^6^. An alternative approach is provided by purely optical Feshbach resonances (OFRs)^7^, which use a light field to couple a colliding atomic pair to a bound molecular state. While an OFR offers great spatial and temporal modulation capabilities, they typically introduce strong losses through the decay of the excited state. First experiments were therefore limited to time scales below 100 μs^8^. Experiments on millisecond time scales have become possible using dipole forbidden intercombination lines, which possess long lifetimes, but which are only available in certain atomic species^9–11^.

Recently, ultra-long-range Rydberg molecules have been proposed as candidates for OFRs^12^. These molecular states are formed by the contact interaction of a quasi-free electron in a Rydberg state with a ground-state atom^13^. Ultra-long-range Rydberg molecules are available for many atomic species and possess long lifetimes which are inherited from the underlying atomic Rydberg excitation.

**Fig. 1:**
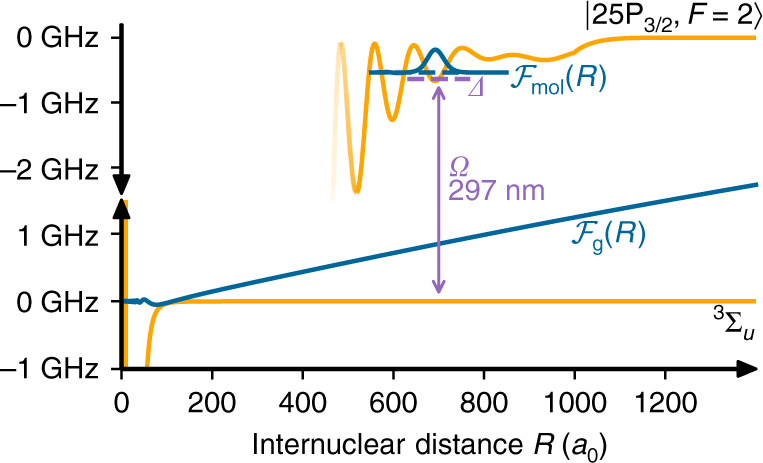
Schematics of the Rydberg optical Feshbach resonance. Two ^87^Rb atoms trapped in a 3D optical lattice scatter in the ^3^Σ_*u*_ channel and are coupled with Rabi frequency *Ω* and detuning *Δ* close to the photoassociation resonance of an ultra-long-range Rydberg molecule in the so-called deep potential, which is adiabatically connected to the ${25{\mathrm{P}}_{3/2},F = 2}$ state. The blue lines denote the relative wave function of the two ground-state atoms ${\cal F}_{\mathrm{g}}(R)$ and the molecular wave function ${\cal F}_{{\mathrm{mol}}}(R)$. Due to the large size of the Rydberg molecule, the coupling takes place at a very large internuclear distance, making it independent of the short-range physics of the open channel (small oscillations of ${\cal F}_{\mathrm{g}}(R)$ for *R* < 100*a*_0_)

The short-range molecular potential between two ground-state atoms gives rise to a phase shift in the relative wave function upon a collision. At ultralow temperature, this can be cast in a single parameter, the s-wave scattering length *a*. In an OFR, the two incoming atoms (open channel) are coupled to a bound molecular state (closed channel) by a laser field. This causes a distortion of the wave function leading to a change in the scattering length and thus a change in the interaction of the two atoms. Depending on the detuning from the molecular resonance, the scattering length can be increased or decreased. The basic principle is sketched in Fig. 1, where we show the relative wave function of two ground-state atoms and the molecular wave function. One peculiarity of a Rydberg OFR is the separation of length scales: the interatomic distance at which the molecule is formed exceeds by far the short-range molecular potential of two ground-state atoms. This has two consequences: first, the coupling strength of the Rydberg OFR is independent of the microscopic details of the open channel, and second the Franck–Condon overlap with the molecular state is much larger compared to previously studied OFRs.

In this work we specifically investigate ^87^Rb atoms colliding in the ^3^Σ_*u*_ ground-state potential which is optically coupled to the vibrational ground state of a molecular state at a bond length of ≈700*a*_0_ (Bohr radius) of the so-called deep ultra-long-range Rydberg molecule potential, which adiabatically connects to the ${25{\mathrm{P}}_{3/2},F = 2}$ state. The binding energy of the molecular state is about *h* × 500 MHz. For details of the calculation and classification of the molecular potential curves, see refs. ^14,15^. To characterize the change in interaction strength we investigate the collapse and revival of the matter wave field of a Bose–Einstein condensate trapped in an optical lattice potential^16^. In the experiment, we start with a superfluid condensate of rubidium atoms in a 3D optical lattice. We then quench the optical lattice deep in the Mott insulating regime, such that the tunneling is frozen out. The matter wave field then undergoes collapse and revival dynamics, where the contrast of the interference pattern is restored after the characteristic time *T* = *h*/*U*, where *U* is the on-site interaction energy in the lattice. The details of the experimental setup can be found in ref. ^17^ and a description of the experimental sequence is given in the methods.

## Results

### Coherent control

**Fig. 2:**
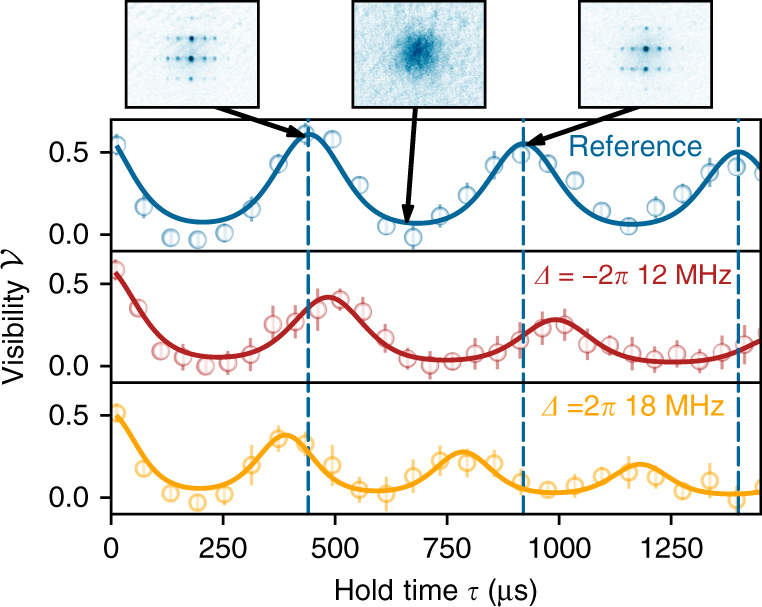
Collapse and revival measurements. After a variable hold time *τ* in the optical lattice, time-of-flight images of the cloud are taken and the visibility of the interference pattern is extracted (see Methods). The blue data correspond to an uncoupled system, the red data to a detuning of *Δ* = −2π × 12 MHz and the yellow data to *Δ* = 2π × 18 MHz (both with atomic Rabi frequency 2*π* × 810 kHz). If not otherwise stated all measurements are taken with a final lattice depth of 30 E_rec_ (recoil energy). An increased (decreased) oscillation frequency and thus interaction strength can be seen for blue (red) detuning. For each hold time we typically record six independent measurements. The error bars denote the statistical error from averaging individual runs

In Fig. 2, we show the time evolution of the visibility ${\cal V}$ of the interference pattern after the quench. From a fit with a model function we extract the corresponding revival time and hence the on-site interaction *U* (see Methods). The upper graph shows the collapse and revival in the absence of any coupling laser and serves as a reference, *U*_ref_. We repeat the experiment, coupling the atoms with a small detuning *Δ* to the photoassociation resonance during the collapse and revival dynamics using a single-photon transition at 297 nm (50 mW power, 100-μm waist, estimated linewidth 200 kHz). In Fig. 2, it is clearly visible that the revival time can be enlarged for red detuning (*Δ* < 0, red curve) and reduced for blue detuning (*Δ* > 0, yellow curve). We then either extract the interaction shift as Δ*U* = *U* − *U*_ref_ or, as the on-site interaction linearly depends on the scattering length, directly calculate the scattering length using

1 $$a = \frac{U}{{U_{{\mathrm{ref}}}}}a_{{\mathrm{bg}}} = \left( {\frac{{{\mathrm{\Delta }}U}}{{U_{{\mathrm{ref}}}}} + 1} \right)a_{{\mathrm{bg}}},$$

with *a*_bg_ = 99*a*_0_ being the background scattering length for rubidium^18^. Our measurements show that the OFR preserves the coherence during the collapse and revival of the many-body wave functions and is therefore a direct demonstration of interaction tuning in a quantum many-body system.

**Fig. 3:**
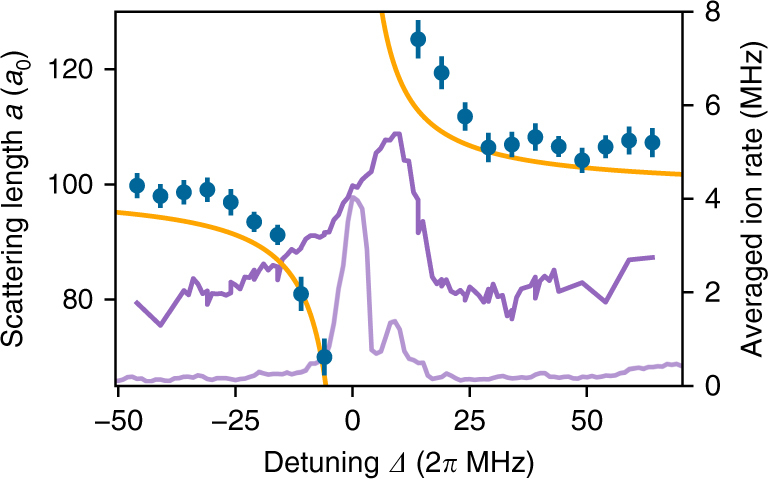
Rydberg optical Feshbach resonance. Measured scattering length (blue dots) for different detunings *Δ* around the photoassociation resonance to the Rydberg molecule shown in Fig. 1, for an atomic Rabi frequency of 2*π* × 810 kHz. As a reference the on-site interaction for the uncoupled system is *U*_ref_ = *h* × 2110(20) Hz. A change of scattering length of ±30*a*_0_ thus corresponds to an interaction shift of about ±600 Hz. The depicted resonances are the averaged ion rate (right scale) within the same measurement procedure over a hold time of *τ* = 450 μs and the same driving strength (purple) and with a reduced Rabi frequency of 2*π* × 230 kHz (light purple). The orange curve is a simplified theoretical model using the experimentally measured atomic Rabi frequency and resonance position (see text). Error bars correspond to statistical errors from the fitting procedure

Varying the detuning or the laser intensity, we can map out the Rydberg OFR. The measured scattering length according to Eq. (1) around the photoassociation resonance to the Rydberg molecule is shown in Fig. 3. The data show the resonance structure expected for an OFR with increasing (decreasing) interaction for blue (red) detuning. We achieve a maximum change in scattering length of ±30*a*_0_ for the selected molecular Rydberg state.

During the coupling to the molecular Rydberg state, we extract ions generated from atomic or molecular Rydberg excitations^19,20^ with a small electric field and record a molecular spectrum (Fig. 3). From the signal for small driving (light purple data in Fig. 3) we can see that the resonance splits into two separate lines. The natural lifetime of the two resonances (measured with a pulsed excitation scheme^20^) is 13.2(6) μs (left peak) and 8.0(4) μs (right peak). This is of the same order as the lifetime of the underlying atomic Rydberg state. From our calculation of the Born–Oppenheimer potentials (see Supplementary Note 1) we do not expect any other deeply bound resonances in the energetic vicinity of the ground state, so that we conclude that both lines correspond to the ground state in the well at ≈700*a*_0_. We believe that the splitting is due to spin-orbit couplings in the p-wave scattering as reported in refs. ^21,22^ which we do not take into account in our potential curve calculations.

For the theoretical description of the OFR we ignore this splitting and treat it as an effective two-level system in the limit of large detuning *Δ*. The detuning is also much larger than any atomic or molecular level shift due to the ponderomotive potentials and the dipole potentials induced by the laser fields. The interaction shift is thus given by ${\mathrm{\Delta }}U = \hbar { {\Omega }}_{{\mathrm{mol}}}^2{\mathrm{/}}(4{ {\Delta }})$. We deduce the molecular Rabi frequency *Ω*_mol_ from the atomic one using ${ {\Omega }}_{{\mathrm{mol}}} = \sqrt 2 FC \times { {\Omega }}$. The atomic Rabi frequency *Ω* is experimentally determined in a separate experiment, the factor $\sqrt 2$ accounts for the symmetry of the excited state, and the Franck–Condon factor *FC* is calculated numerically (see Supplementary Notes 2–4). For a lattice depth of 30 E_rec_ we find *FC* = 0.12 corresponding to a molecular Rabi frequency of *Ω*_mol_ = 2*π* × 140 kHz for *Ω* = 2*π* × 810 kHz. We set the resonance position to be at the center of the stronger molecular peak at small driving. The model has no adjustable parameters, and, despite the simplifications, it shows qualitatively good agreement with the measured data in Fig. 3. Our treatment as a bound–bound transition in an effective two-level system is equivalent to the usual treatment as a free-bound transition provided the size of the molecule lies within the linear part of the relative wave function of the two ground-state atoms (see Supplementary Note 5). For a more rigorous treatment of Rydberg OFR using scattering theory, we refer to ref. ^12^.

### Dissipative effects

The dispersive nature of an OFR is inevitably accompanied with absorption. As in other experiments with OFRs^9,10,23^, we observe losses beyond the expected power broadening (purple data in Fig. 3). However, in a continuously driven Rydberg system, the excitation dynamics are even more complex. Broadening from black-body-induced transitions as reported in ref. ^24^, as well as correlated cluster dynamics^25–28^ will play an important role in the observed line shape on time scales in the millisecond range. Also the interaction with ions cannot be excluded, as they are continuously created in the sample and stay in the cloud for about 1 μs. All these inhomogeneous broadening effects contribute to the linewidth and the background signal. Thereby, the observed linewidth of about 20 MHz is of the same order of magnitude as the broadening of atomic Rydberg resonances for similar Rabi frequencies and atomic densities^24^. Despite the large broadening effects, the lifetime of the sample is still in the order of 1 ms, which is of similar performance as previous OFR experiments using intercombination lines^9,10^. As we will discuss later on, this does not constitute a fundamental limit to a Rydberg OFR, but is rather due to technical limitations and the particular implementation in our system.

### Interaction effects

We close this work by discussing a conceptually new aspect of a Rydberg OFR, which is absent in other Feshbach resonance experiments. This is the back action of the dissipative effects on the dispersive part of the resonance. Due to the long-range interaction, the presence of atomic and molecular Rydberg excitations, which are continuously created by the dissipative effects discussed above, leads to an interaction induced shift of the molecular resonance. The van der Waals coefficient is positive for the 25P_3/2_ state of rubidium. Consequently, we expect a blue shift of the molecular resonance which scales as ${ {\Delta }}_{{\mathrm{Ryd}}} = C_6{\mathrm{/}}r_{{\mathrm{Ryd}}}^6$ = 4 MHz × μm^6^/$r_{{\mathrm{Ryd}}}^6$ if a Rydberg excitation is present at distance *r*_Ryd_. Because the Rydberg molecule wave function is dominantly composed by the atomic 25P_3/2_ state, the *C*_6_ coefficient of the Rydberg molecule is that of the Rydberg atom. The number of Rydberg excitations simultaneously present in the system can be deduced from the measured ion rate (2–3 MHz) and amounts to 200–300 (see Methods). For our trap geometry (almost isotropic atomic cloud with a Thomas–Fermi radius of *r*_TF_ ≈ 10 μm), we estimate an average distance of about 1 μm between an atom and the nearest Rydberg excitation, leading to a blue shift of the molecular resonance of a few MHz.

In addition, the DC Stark effect from the ions created by photoionization^19^ and associative ionization^20^ of Rydberg atoms and molecules leads to an opposite effect. For the 25P_3/2_ state, a red shift of the molecular resonance occurs, ${ {\Delta }}_{{\mathrm{ion}}} \approx - 207$ MHz × μm^4^/$r_{{\mathrm{ion}}}^4$, where *r*_ion_ denotes the average distance to the nearest ion. Due to the applied electric field, the ions leave the cloud in about 1 μs and we estimate on average 2–3 ions being simultaneously present in the cloud, leading to *r*_ion_ = 5–10 μm.

**Fig. 4:**
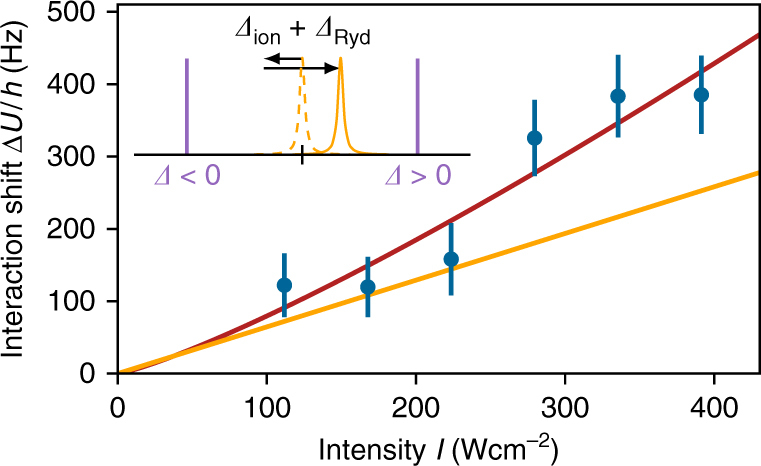
Intensity-dependent interaction shift. Measured interaction shift Δ*U*, for different coupling laser intensities and a detuning *Δ* = 2*π* × 18 MHz. The data point at ≈340 W cm^−2^ corresponds to an atomic Rabi frequency of *Ω* = 2*π* × 810 kHz as in Fig. 3. The data fits well to the two-level model for low intensities (orange); however, for strong driving, we observe an increase of the interaction. The red line is a power law fit *I*^*α*^, which yields an exponent of *α* = 1.2(3). Error bars correspond to statistical errors from the fitting procedure. The inset illustrates the back action of the dissipative effects on the Rydberg OFR. Ions shift the molecular resonance to the red, while Rydberg excitations lead to a blue shift. The dashed yellow line denotes the unperturbed resonance and the solid yellow line denotes the shifted resonance. Depending on the laser detuning *Δ* (sketched as purple lines), the effective detuning from the resonance is either enlarged (*Δ* < 0) or reduced (*Δ* > 0)

The effective interaction shift is then given as ${\mathrm{\Delta }}U = \hbar {\mathrm{\Omega }}_{{\mathrm{mol}}}^2{\mathrm{/}}(4{ {\Delta }}_{{\mathrm{eff}}})$, where *Δ*_eff_ = *Δ* + *Δ*_ion_ + *Δ*_Ryd_. In our particular situation, we expect an overall blue shift of the resonance as the van der Waals interaction dominates. This is illustrated in the inset of Fig. 4. Ions in the sample lead to a red shift of the resonance, which is over compensated by the shift through Rydberg excitations. For excitation light blue detuned to the unperturbed resonance we thus expect a smaller effective detuning and for red detuned an increased effective detuning. Note that the same effect might also explain the observed blue shift of the resonance position for strong driving in Fig. 3, which we did not discuss so far.

We have performed two experiments to find indications for this effect. In Fig. 4, we show the intensity dependence of the interaction shift for *Δ* > 0 (blue detuning). Indeed, we find an increased interaction shift for larger intensities as compared to the two-level model. A power law fit (red line in Fig. 4) yields an exponent of *α* = 1.2(3), and does therefore not allow to draw reliable conclusions on the magnitude of the nonlinearity.

**Fig. 5:**
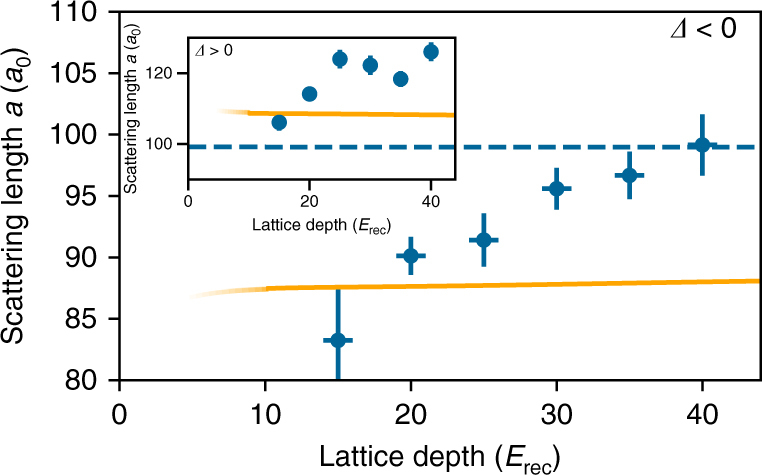
Interaction effects of a Rydberg optical Feshbach resonance. Dependence of the measured scattering length *a* = *U*/*U*_ref_ *a*_bg_ on the final lattice depth for red detuning *Δ* = −2*π* × 17 MHz (inset for blue detuning *Δ* = 2*π* × 18 MHz). While our two-level theory (orange) has a near constant behavior the experimental scattering length increases with increasing lattice depth for both blue and red detuning. We attribute this to background Rydberg excitations leading to a shift of the photoassociation resonance (see text). Error bars in the scattering length correspond to the statistical errors from the fitting procedure. Error bars in the lattice depth (±1 *E*_rec_) are an estimated systematic error

Much clearer evidence for the resonance shift can be found by varying the lattice depth for a given laser intensity and detuning. An increase in lattice depth causes a larger Franck–Condon overlap as the Wannier function is compressed. This leads to an increase in the interaction shift. At the same time, the on-site interaction energy *U*_ref_ stemming from the background scattering length scales in the same way, such that the measured scattering length should remain unchanged. The dependence on the lattice depth therefore measures the influence of the Rydberg population on the scattering length for otherwise identical parameters of the OFR. In Fig. 5, we show the change in scattering length for red as well as blue detuning for different lattice depths. For red detuning the scattering length increases with increasing lattice depth until it approaches the background scattering length of 99*a*_0_. For blue detuning, an increase of scattering length can be observed, in accordance with a decreased effective detuning, while for the largest lattice depths the effect seems to saturate. This might be explained by anti-blockade effects in the system (the facilitation radius is about 1 μm), which set an upper limit for the maximum possible Rydberg density. The interaction-induced nonlinearity in the system might also explain the previously not discussed variations seen in Fig. 3, where the measured data tends to be above the theoretical model for all detunings.

## Discussion

The explanation given above is capable of qualitatively describing our experimental findings. A true quantitative evaluation of the effect is, however, challenging and is not within the scope of this work. Such a microscopic model would have to include the microscopic positions of the Rydberg excitations and the ions as well as their mutual acceleration and spatial trajectories. Rydberg blockade effects have to be considered as well as the discrete underlying lattice structure and the trap geometry. Moreover, dipole allowed black-body-induced transitions into neighboring Rydberg states lead to an additional resonant dipole–dipole interaction, scaling as *r*^−3^.

Rydberg OFRs have a couple of advantages in comparison to previously observed OFR. First, they are readily available in many atomic species. Rydberg molecules have been experimentally measured in rubidium^13^, cesium^29^, and strontium^30^, and they are expected to exist for all species or atomic mixtures having a negative electron-ground state scattering length^12^. As stated in ref. ^31^ this is the case for the triplet scattering length in all alkali atoms. Second, the molecular states can be addressed spin-resolved and even two OFR can be active at the same time. Most importantly, however, we believe a Rydberg OFR is not limited by the performance shown here. Note that our preparation scheme provides rather high atomic densities and the average occupation number in the center of the cloud is estimated to be about 6–8 atoms per lattice site. Our experiment is therefore particularly sensitive to density-dependent losses. Thus, reducing the atomic density in future experiments to an average filling factor of $\overline n = 1$ should significantly reduce these losses. Furthermore, the coupling laser intensity could be increased by a factor of 10 compared to the measurements reported here, using commercially available high power UV laser systems. Additionally, Rydberg molecules feature a plenitude of available states with long lifetimes. For a state bound at ≈550*a*_0_ in the same potential we have already measured a change in scattering length of ≈5*a*_0_, while maintaining a lifetime of 25 ms. The product of these two numbers defines a figure of merit to characterize a resonance. Thus, this already corresponds to an increase in performance by a factor of five compared to the discussed resonance in this article. We believe an even better performance can be achieved by using states with lower principle quantum number *n*. While these have a reduced lifetime ∝*n*^3^, the interaction shift due to a stronger coupling scales as ∝*n*^−3^. Because of a deeper binding energy ∝*n*^−6^ and a reduced van der Waals coefficient ∝*n*^11^ for Rydberg–Rydberg interactions^32^, it should be possible to operate the OFR outside the spectral region of interaction-induced broadening. If that was accomplished, Rydberg OFR could become competitive to magnetic Feshbach resonances, with the additional advantage of combining fast temporal modulations of the interaction on short-length scales. Coupling to more exotic molecular states such as the recently discovered butterfly or trilobite molecules^29,33^, possessing permanent electric dipole moments up to several kilodebye, could even be a way to tune anisotropic interactions via the p-wave scattering length.

There is one more aspect, which makes Rydberg OFR truly unique and appealing. This is the availability of molecular trimer or tetramer states^34^. Coupling an atomic gas to such a molecule allows to engineer genuine attractive or repulsive three-body and four-body interactions in the gas. Such experiments would open up a so far unexplored territory for interacting many-body systems.

## Methods

### Experimental procedure

To perform our experiments we prepare a Bose–Einstein condensate of ≈80 × 10^3^ ^87^Rb atoms, spin polarized in the $\left| {5{\mathrm{S}}_{1/2},F = 2,m_F = 2} \right\rangle$ state, in a crossed optical dipole trap with trapping frequencies *ω*_*x*,*y*,*z*_ = 2*π* × (61, 61, 51) Hz. We adiabatically transfer these atoms into a blue detuned 3D optical lattice with lattice constants *a*_*x*,*y*,*z*_ = (374, 374, 529) nm and lattice depth of *S* = 8 E_rec_ with an exponential ramp with time constant 20 ms. The lattice depth is then linearly increased within 50 μs to a final depth of *S* = 30 E_rec_, while simultaneously switching off the underlying harmonic trapping potential. The coupling light at 297 nm is generated from a frequency doubled dye laser. The stated atomic Rabi frequencies are calibrated from a different set of measurements (see Supplementary Note 1). It is linearly polarized parallel to the quantization axis of the system. We ramp it to its final power within the same 50 μs while we are increasing the lattice depth. After a variable hold time *τ*, we switch-off the lattice potential as well as the coupling laser instantaneously and take a time-of-flight image of the dropping atomic cloud. Additionally we record the time resolved ion signal during illumination using a small electric field (*E* ≈ 0.2 V cm^−1^). Ions are created from excited Rydberg states mainly through photoionization from the lattice beams at a small rate (≈5 kHz) compared to the natural decay or the coupling strength. From the measured ion rate we can estimate the number of excitations in the sample. As the natural decay occurs with ≈50 kHz only every tenth excitation is ionized. Thus, for a measured ion rate of 1 MHz, one excitation is created in the system with 10 MHz or every 100 ns. As each excitation lives ≈10 μs we have about 100 excitations in the system at any given time for an ion rate of 1 MHz.

### Determination of the on-site interaction

As we switch-off the underlying trapping potential, the collapse and revival dynamics are on top of Bloch oscillations in the lattice potential. Thus, to extract the visibility ${\cal V}$ of the interference pattern, for a given hold time *τ*, we extract the center of the atomic cloud from a time-of-flight image. From this central peak we take the usual approach defining four boxes around the center containing the super-fluid peaks, as well as four boxes turned by 45° with respect to the first four boxes^35^. We define the visibility ${\cal V}$ as the pixel sum from the first set of boxes minus the pixel sum of the second set of boxes divided by the pixel sum of all eight. A super fluid state would thus have a visibility equal to one and a collapsed state equal to zero.

To extract the on-site interaction *U* we fit the measured visibility using:

2 $$\begin{array}{*{20}{l}} {\cal V} \hfill & \propto \hfill & {\left| {\left\langle {\alpha (t)} \right|\hat a\left| {\alpha (t)} \right\rangle } \right|^2{\mathrm{e}}^{ - \tau /\tau _0}} \hfill \\ {} \hfill & = \hfill & {\left| {\sqrt {\overline n } {\kern 1pt} {\mathrm{exp}}\left( {\overline n \left( {{\mathrm{e}}^{i( - U/\hbar {\kern 1pt} \tau + \varphi )} - 1} \right)} \right)} \right|^2{\mathrm{e}}^{ - \tau /\tau _0}.} \hfill \end{array}$$

The overall exponential decay factor ${\mathrm{e}}^{ - \tau /\tau _0}$ accounts for any imperfections and losses in the system and depends on the detuning and driving strength of the coupling laser. The decay time *τ*_0_ shows a similar behavior as the resonance structures depicted in Fig. 3 varying from about 2 ms away from the resonance to 800 μs for the points close to the resonance. $\overline n$ is the average atom number per site and *ϕ* a phase offset caused by the finite ramp length of the lattice depth. For all measurements we simultaneously record a reference measurement without coupling to the molecular state to compensate for day to day drifts and alignment imperfections in the lattice beams and extract the interaction shift Δ*U* = *U* − *U*_ref_.

### Data availability

The data that support the findings of this study are available from the corresponding author upon reasonable request.

## Electronic supplementary material

Supplementary Information
