# Supplementary material for: Experimental realization of a Rydberg optical Feshbach resonance in a quantum many-body system
Source: Nat Commun. 2018 Jun 8;9:2238. doi: 10.1038/s41467-018-04684-w (PMC5993778; doi:10.1038/s41467-018-04684-w)
Supplement: Supplementary file 1 — Supplementary Information [file 41467_2018_4684_MOESM1_ESM.pdf]

Supplementary Material: Experimental realization of a Rydberg optical Feshbach resonance in a quantum many-body system

O. Thomas,[1,2] C. Lippe,[1] T. Eichert,[1] and H. Ott[1,*]

[1] *Department of Physics and Research Center OPTIMAS, Erwin-Schrdinger-Strae 46, Technische Universitt Kaiserslautern, 67663 Kaiserslautern, Germany*

[2] *Graduate School Materials Science in Mainz, Staudinger Weg 9, 55128 Mainz, Germany*

SUPPLEMENTARY NOTE 1: MOLECULAR STATES

To calculate the potential energy curves we follow the procedure from our previous work described in [1] including s- and p-wave scattering as well as the ground state hyperfine interaction. This procedure yields the potential energy curves $U_{\mathrm{BO}}(R)$ in Born Oppenheimer approximation. The molecular wave functions are then solutions of the radial Schrödinger equation:

$$\left(-\frac{\hbar^2}{2\mu}\frac{\partial^2}{\partial R^2}+U_{\mathrm{BO}}(R)\right)\mathscr{F}_{\mathrm{mol}}(R)=E_{\mathrm{bond}}\mathscr{F}_{\mathrm{mol}}(R), \tag{1}$$

with μ the reduced mass of the two nuclei. We solve supplementary equation (1) numerically by integrating the radial wave function using a numerov method and determining the bound state energies with a shooting method [2].

SUPPLEMENTARY NOTE 2: RABI FREQUENCY CALIBRATION

To calibrate the Rabi frequency Ω of the excitation system we measure the light shift from the excitation light on the $|F=2,m_F=+2\rangle$ ground state. We prepare an atomic cloud in the $|F=1,m_F=+1\rangle$ state and illuminate it with the excitation light blue detuned to the $|F=2,m_F=+2\rangle$ to $|25\mathrm{P}_{3/2}\rangle$ transition while probing the lower transition with a microwave pulse. From the resonance positions of the $|F=2,m_F=+2\rangle$ state for different intensities and detuning Δ (in the range of 50 to 200 MHz) of the excitation light we calibrate the Rabi frequency to the excited state, using the shift in a 2-level system for large detunings, as $\Delta E=\hbar\frac{\Omega^2}{4\Delta}$.

SUPPLEMENTARY NOTE 3: FRANCK-CONDON FACTOR

To calculate the Franck-Condon factor for a photoassociation resonance we approximate the ground state wave function with the ground state of an isotropic harmonic oscillator not taking into account any interaction effects between two atoms in a lattice site. We use the geometric mean of the trapping frequencies in the different lattice axes. We can then analytically write the relative ground state wave function of two atoms as:

$$\begin{aligned}\Psi_g(R,\Theta,\Phi)&=Y_{00}(\Theta,\Phi)\mathscr{F}_g(R)/R\\&=\frac{1}{\sqrt{4\pi}}\frac{2}{\pi^{1/4}}\left(\frac{\mu\omega}{\hbar}\right)^{3/4}\mathrm{e}^{-\frac{\mu\omega}{2\hbar}R^2}.\end{aligned}\tag{2}$$

Using this and the numerically integrated molecular nuclei wave function $\mathscr{F}_{\mathrm{mol}}(R)$ we evaluate the Franck-Condon factor using

$$FC=\int dR\mathscr{F}^*_{\mathrm{mol}}(R)\mathscr{F}_g(R). \tag{3}$$

SUPPLEMENTARY NOTE 4: SYMMETRY FACTOR

When coupling a lattice site with N atoms to a Rydberg molecule an increase in coupling strength of $\sqrt{N(N-1)}$ is to be expected, because of the symmetry of the excited state. For instance for 3 atoms there are 6 possible excitation configurations. The coupling strength should thus be $\Omega'_{\mathrm{mol}}=\sqrt{N(N-1)}FC\Omega$.

In a collapse and revival experiment the revival time is given as h/U, because the different Fock states evolve in time with eigenenergies $U/2\,N(N-1)$. Adding the shift from the molecular Rydberg states leads to eigenenergies

$$\frac{U_{\mathrm{ref}}}{2}N(N-1)+\frac{\Omega'^2_{\mathrm{mol}}}{4\Delta}=\frac{1}{2}\left(U_{\mathrm{ref}}+\frac{(\sqrt{2}FC\Omega)^2}{4\Delta}\right)N(N-1). \tag{4}$$

The shift through the coupling is thus equal to a 2-level system with a factor of $\sqrt{2}$ as discussed in the main text.

SUPPLEMENTARY NOTE 5: EQUIVALENCE OF BOUND-BOUND AND FREE-BOUND TRANSITIONS IN THE LIMIT OF VANISHING LATTICE POTENTIAL

Bound-Bound: In a simplified two level bound-bound model we can express the change in scattering length as $\Delta a = \frac{\Delta U}{U_0} a_{\mathrm{bg}}$, where ΔU is the shift through the light field $\Delta U \approx \hbar \frac{\Omega_{\mathrm{mol}}^2}{4\Delta}$ (for $\Delta \gg \Omega_{\mathrm{mol}}$), U_0 the unperturbed on-site interaction and a_{bg} the background s-wave scattering length. First we evaluate the on-site interaction for a spherical symmetric lattice site in harmonic approximation. We can then express the Wannier function as a Gaussian of the form:

$$\omega_0(x) = \left(\frac{m\omega}{\pi\hbar}\right)^{1/4} \exp\left(-\frac{m\omega}{2\hbar}x^2\right) \tag{5}$$

where ω is the trapping frequency of the potential. The on-site interaction is then given as

$$\begin{aligned} U_0 &= \frac{4\pi\hbar^2}{m} a_{\mathrm{bg}} \int dxdydz \left|w_0(x)w_0(y)w_0(z)\right|^4 &(6)\\ &= \frac{4\pi\hbar^2}{m} a_{\mathrm{bg}} \left(\frac{m\omega}{2\pi\hbar}\right)^{3/2}. &(7) \end{aligned}$$

To compare the change in scattering length to the free-bound case it is necessary to split the Rabi frequency into several parts. We thus write

$$\Omega_{\mathrm{mol}} = \frac{d_{\mathrm{mol}}E}{\hbar} FC \tag{8}$$

where d_{mol} is the molecular dipole matrix element, E is the electric field strength of the excitation light and FC is the bound-bound Franck-Condon factor. As before the relative ground state wave function is hereby approximated as a Gaussian wave function in relative coordinates

$$\mathscr{F}_g(R) = \frac{2}{\pi^{1/4}} \left(\frac{\mu\omega}{\hbar}\right)^{3/4} R \exp\left(-\frac{\mu\omega}{2\hbar}R^2\right), \tag{9}$$

with $\mu = m/2$ the reduced mass of the two atoms. We ignore any interaction between the two particles leading to a separation of $\approx a_{\mathrm{bg}}$ for $R \to 0$. Putting this together we end up with

$$\begin{aligned} \Delta a &= \frac{\Delta U}{U_0} a_{\mathrm{bg}} \\ &\approx \frac{d_{\mathrm{mol}}^2 E^2}{4\Delta\hbar} \frac{m}{4\pi\hbar^2} \left(\frac{2\pi\hbar}{m\omega}\right)^{3/2} \left|\int \mathscr{F}_{\mathrm{mol}}(R)\mathscr{F}_g(R)dR\right|^2 \\ &= \frac{d_{\mathrm{mol}}^2 E^2 \mu}{2\Delta\hbar^3} \left|\int \mathscr{F}_{\mathrm{mol}}(R) R \exp\left(-\frac{\mu\omega}{2\hbar}R^2\right) dR\right|^2 \\ &\approx \frac{d_{\mathrm{mol}}^2 E^2 \mu}{2\Delta\hbar^3} \left|\int \mathscr{F}_{\mathrm{mol}}(R) R dR\right|^2. \end{aligned} \tag{10}$$

Where in the last step we approximated $\exp\left(-\frac{\mu\omega}{2\hbar}R^2\right) \approx 1$ for $\omega \to 0$.

Free-Bound: For a free-bound transition on the other hand we adapt the method from ref. [3]. Here the interaction shift is given by $\Delta a = l_{\mathrm{opt}} \frac{\Delta\gamma}{\Delta^2+\gamma^2/4}$ with l_{opt} the optical coupling strength and γ the decay from the excited state. We will evaluate this in the limit $\Delta \gg \gamma$ neglecting the second term in the denominator leading to $\Delta a = l_{\mathrm{opt}}\gamma/\Delta$. The optical coupling strength is given by Fermi's golden rule

$$l_{\mathrm{opt}} = \frac{\Gamma(k)}{2k\gamma} = \frac{1}{2k\gamma} \frac{2\pi}{\hbar} \left|\langle n| V_{\mathrm{opt}} |E\rangle\right|^2 \tag{11}$$

where $|n\rangle$ is the normalized excited state and $|E\rangle$ is an energy normalized free state given by

$$\langle R|E\rangle = \sqrt{\frac{2\mu}{\pi\hbar^2 k}} \sin(kR). \tag{12}$$

We again have ignored any interaction effects for $R \to 0$, and V_{opt} is the optical coupling potential. We rewrite

$$\begin{aligned} \langle n| V_{\text{opt}} |E\rangle &= \frac{\hbar\Omega_{\text{mol}}}{2} = \frac{d_{\text{mol}}E}{2} FC \\ &= \frac{d_{\text{mol}}E}{2} \sqrt{\frac{2\mu}{\pi\hbar^2 k}} \int \mathscr{F}_{\text{mol}}(R) \sin(kR) dR \\ &\approx \frac{d_{\text{mol}}E}{2} \sqrt{\frac{2\mu k}{\pi\hbar^2}} \int \mathscr{F}_{\text{mol}}(R) R dR \end{aligned} \tag{13}$$

where the last step is valid for $k \to 0$, as is the case for ultra cold atomic clouds. Putting all this together we end up with

$$\begin{aligned} \Delta a &\approx \frac{1}{2k\Delta} \frac{2\pi}{\hbar} \frac{d_{\text{mol}}^2 E^2}{4} \frac{2\mu k}{\pi\hbar^2} \left| \int \mathscr{F}_{\text{mol}}(R) R dR \right|^2 \\ &= \frac{d_{\text{mol}}^2 E^2 \mu}{2\Delta\hbar^3} \left| \int \mathscr{F}_{\text{mol}}(R) R dR \right|^2 \end{aligned} \tag{14}$$

which is equivalent to supplementary equation (10) for a bound-bound description.

SUPPLEMENTARY REFERENCES

* ott@physik.uni-kl.de
[1] Niederprüm, T., Thomas, O., Eichert, T. & Ott, H. Rydberg Molecule-Induced Remote Spin Flips. Phys. Rev. Lett. **117**, 123002 (2016).
[2] Stoer, J. & Bulirsch, R. Introduction to Numerical Analysis, vol. 12 (Springer Science & Business Media, 2013).
[3] Nicholson, T. L. et al. Optical Feshbach resonances: Field-dressed theory and comparison with experiments. Phys. Rev. A **92**, 022709 (2015).
